# Comparative analysis of bone outcomes between quantitative ultrasound and dual-energy x-ray absorptiometry from the UK Biobank cohort

**DOI:** 10.1007/s11657-023-01287-x

**Published:** 2023-05-30

**Authors:** Paul A Swinton, Kirsty J Elliott-Sale, Craig Sale

**Affiliations:** 1 School of Health Sciences, Robert Gordon University, Garthdee Road, Aberdeen, AB10 7QE UK; 2 Department of Sport and Exercise Sciences, Institute of Sport, Manchester Metropolitan University, M1 7EL, Manchester, UK

**Keywords:** Reliability, Criterion validity, Osteoporosis, Osteopenia, Heel ultrasound

## Abstract

***Summary*:**

This large cohort study investigated reliability and validity of heel ultrasound to estimate bone mineral density in adults. Reliability calculated between left and right heels was relatively poor and so was criterion validity assessed relative to dual-energy X-ray absorptiometry. Heel ultrasound should be used cautiously when estimating bone mineral density.

**Introduction:**

Calcaneal quantitative ultrasound (QUS) may be used as a safe, low cost, and portable means to estimate bone mineral density (BMD) in large cohorts. The purpose of this study was to quantify the reliability and validity of QUS in comparison to dual-energy X-ray absorptiometry (DXA), which is the reference method for BMD measurement and diagnoses of osteopenia and osteoporosis.

**Methods:**

Bone outcomes measured on the large UK Biobank cohort were used. The reliability of QUS estimated BMD was quantified by comparing values obtained from the left and right heel measured in the same session. Criterion validity was assessed through agreement between QUS and DXA, quantifying correlations, and sensitivity and specificity of osteopenia and osteoporosis diagnoses.

**Results:**

Reliability calculations were made using data from over 216,000 participants demonstrating similar QUS BMD values between left and right heels in the absolute scale (Sd of difference for men: 0.12 and 0.07 g·cm^−2^). However, when expressed in relative scales, including concordance of quartiles, reliability was poor. Agreement between QUS and DXA was quantified using data from 5042 participants. Low to modest correlations (*r* = 0.29 to 0.44) were obtained between multiple QUS variables and DXA BMD, with sensitivity identified as very poor (0.05 to 0.23) for osteoporosis, and poor (0.37 to 0.62) for osteopenia diagnoses.

**Conclusions:**

The findings of this large comparative analysis identify that whilst calcaneal QUS has the potential to produce reliable absolute BMD measurements and demonstrate modest associations with DXA BMD measures, use of that information to make relative statements about participants in the context of the larger population or to appropriately diagnose osteopenia or osteoporosis may be severely limited.

## Introduction

As the elderly population expands, increased health and social costs are expected with greater instances of conditions associated with low bone mass (e.g., osteopenia and osteoporosis). Osteoporosis is the most common age-associated disease of the musculoskeletal system, with over 22 million women and 5.5 million men in the European Union estimated to have this health condition [1]. The major clinical consequence of osteoporosis includes fragility fractures, which affect around 35% of women and 17% of men in the United Kingdom (UK) and the rest of Europe [2]. Not only is there a large health burden of osteoporosis, but the associated economic burden of fragility fractures is significant, with a recent review estimating associated costs of $17.9 billion and £4 billion per annum in North America and the UK [3].

Diagnostic criteria for osteopenia and osteoporosis were developed by the World Health Organisation [4], leading to operational definitions based upon standardized bone mineral density (BMD) assessments (T scores) at various skeletal sites. These criteria include a BMD T-score between −1.0 and −2.5 diagnosing osteopenia, and a T score of −2.5 or lower diagnosing osteoporosis [4]. Currently, the criterion method for BMD measurement is dual energy X-ray absorptiometry (DXA), with diagnostic confirmation preferably sought prior to any pharmacological intervention. BMD measurement using DXA may not, however, be as widely available as desired due to the high cost of the equipment, the need for supported resources (including trained operators), and the need for a relatively permanent location due to limited transportability. Additionally, DXA omits a dose of ionising radiation, such that recommendations are that its use must be kept as low as reasonably achievable [5]. Given the limiting factors of DXA and the need to screen individuals worldwide often in rural and resource-constrained areas [6] to prevent large scale underdiagnosis of osteoporosis, other technologies have been developed with the hope they can provide suitable estimates of BMD without the associated costs, resource implications, and potential harms. One such technology includes quantitative ultrasound (QUS), which provides a non-invasive method to estimate BMD and other potentially relevant bone structural characteristics at peripheral skeletal sites [7]. There are several types of QUS devices available (for a review, please see [8]), with each measuring the velocity of transmission and amplitude of the ultrasound signal at specific skeletal sites, such as the hand or the heel, but also less commonly the tibia [7]. The broadband ultrasound attenuation (BUA) measured by QUS is influenced by the density, architecture, and elasticity of the bone tissue (for a review, please see [9, 10]).

Whilst the potential benefits of using QUS in terms of its cost and accessibility, particularly in paediatric populations and in areas with lower socioeconomic status, are clear, no direct measurement of bone mass is made. Systematic reviews have identified an association between QUS derived measurements and risk of overall fragility fracture [11, 12], although there remain concerns regarding the precision of QUS, with measurement accuracy known to be highly susceptible to factors such as the thickness of the overlying tissue and orientation of the probe [13]. In addition, comparative analyses of bone outcomes between QUS and DXA (as the reference) have produced inconsistent findings. To better assess the suitability of QUS to measure and screen BMD, large scale studies which are at present still limited, are required across different populations to accurately quantify reliability and agreement. Recently, Nguyen et al. [14] quantified associations between calcaneal QUS and DXA in 1270 women and 773 men as part of the Vietnam Osteoporosis Study. Only modest correlations were obtained between BUA and BMD measured at the lumbar spine (*r* = 0.34) and femoral neck (*r* = 0.35), leading the authors to conclude that QUS was limited and unlikely to be suitable for osteoporosis screening. In a much smaller scale study, Weeks et al. [15] compared calcaneal BUA and DXA measurements of bone mass in 389 children aged between 4 and 18 years. Weeks et al. [15] also reported modest correlations (*r* = 0.46 to 0.54) with poor agreement obtained between quartile rankings from the different measurements (27.3 to 38.2%). The conclusions of Weeks et al. [15] and Nguyen et al. [14] that QUS is not an appropriate tool for screening is in contrast to other authors that state QUS should be considered an accurate diagnostic tool, at least for certain populations such as postmenopausal women [16]. More recent recommendations have called for QUS to be used as a pre-screening tool to reduce the number of DXA screenings required [17]. Given divergent opinions on the use of QUS, there is still a need for further research, including large scale studies that seek to investigate the factors that may influence the appropriateness of QUS. One area that has received limited study is the reliability of QUS and factors that may influence measurement error. Herein, we aimed to further explore the potential utility of BUA estimations of BMD by first quantifying reliability and secondly by quantifying agreement with measures of BMD taken from DXA in a large sample. To do this, we made use of data available through the UK Biobank, which allowed for exploration of these questions in a very large population cohort.

## Method

### Participants

The UK Biobank is a large population cohort study that recruited more than 500,000 participants registered with the National Health Service in the UK between 2007 and 2010. Participants were aged between 40 and 69 years and undertook baseline assessments at 22 centres across England, Scotland, and Wales. A range of demographic, lifestyle, and medical data were collected, including imaging and musculoskeletal assessments, with full protocols publicly available (UK [18]). The UK Biobank protocol complied with the Declaration of Helsinki and was approved by the North-West Multi-Centre Research Ethics Committee. Participants provided their informed consent before taking part.

### Study procedures

QUS of the heel was performed using the Sahara Clinical Sonometer (Hologic, Bedford, Massachusetts) according to a standardised protocol (UK [19]). Prior to assessment, trained staff checked if participants were able to undertake both left and right heel measurements. Those with open wounds, breaks or sores around the heel, or metal parts (such as pins) in the heel did not undertake the related measurement. Each centre used the same machine model, and quality control was performed daily using a phantom, as per manufacturer’s instructions. Use of the device generates two variables, including speed of sound (SOS) and BUA. SOS measures the speed at which ultrasound travels through bone and is calculated by dividing the ultrasound transit time by the length of body part. Whereas BUA is the slope between the attenuation of the sound signal and its frequency as it travels through the bone and soft tissue, with greater bone health described by both higher SOS and BUA values. Heel BMD was measured using the following formula: BMD = 0.002592 ×(BUA+SOS)−3.687. Sex-specific BMD T scores were calculated as the number of standard deviations the Heel BMD was above or below the standard (UK [19]). Sex-specific BMD Z scores were also calculated in reference to the mean and standard deviation values obtained from the current sample.

DXA data were collected from approximately 5000 participants between 2014 and 2015 (UK [20]). The iDXA instrument was calibrated to the manufacturer’s phantom (GE-Lunar, Madison, WI) and underwent a daily quality control procedure. Whole body scans were made with the participant asked to lie flat on their back on the DXA couch. Scans of the lumbar spine were made with participants lower legs placed on a polystyrene block, bringing the hips and knees to 80° of flexion. Scans of the femur were made with the participants’ leg straight and foot strapped against a support to ensure correct orientation of the hip. The scans were subsequently analysed by a qualified radiographer at, or soon after, their acquisition in order to generate numerical measures of bone area, BMD and BMC (among other outcomes, see UK [20]) at each of the relevant sites. For the purposes of the current study, we made use of BMD data obtained from the sites most common to assess an individual’s bone health including the whole body, lumbar spine and femoral neck. Sex-specific DXA BMD T scores and Z scores were calculated based on the number of standard deviations above or below the standard and the current sample mean, respectively.

### Statistical analyses

Reliability of QUS was assessed by comparing BMD, BMD T scores and BMD Z scores obtained from the left and the right heel within the same measurement session. This measure of reliability reflects the limitations of the retrospective analysis where ideally reliability would be assessed using repeated measurements on the same heel. Left to right comparison was therefore viewed as an upper bound of intra-session reliability. No attempts were made to remove outliers, such that the reliability assessment captured the overall variability in measurements obtained. Reliability was conceptualised in two forms including [21] the typical variation in magnitude; and [7] the variation in population ranking between measurements obtained within the same session. Specifically, variation in magnitude was assessed by calculating the standard deviation of the difference in values obtained from left and right heels and quantifying the extent to which the value was influenced by sex, age, and the expected value. This was achieved using univariate Gaussian distributional regression models, with sex, age, and mean of left and right values used as inputs to the model. Age and mean values were centred and scaled by dividing by two times the sample standard deviation to establish which factor was most influential in influencing reliability [22]. Variation in population ranking was assessed by placing participants into sex-specific quartiles and quantifying the concordance of quartile selection from the values obtained from left and right heels.

Criterion validity was assessed by comparing all QUS variables (SOS, BUA, BMD, BMD T scores, and BMD Z scores) with BMD DXA values obtained from the total body, lumbar spine and femoral neck. Criterion validity was conceptualised in three forms including [21] overall linear relationships between QUS and DXA values; [7] typical magnitude of difference between QUS and DXA values measured on the same scale (e.g., BMD Z scores); and [3] concordance of osteopenia and osteoporosis diagnoses between QUS and the reference DXA. Overall linear relationships were quantified using sex-specific correlations for each QUS variable with DXA BMD values. Typical magnitude of difference was quantified comparing BMD Z scores obtained with QUS and DXA, using Gaussian distributional regression models investigating the extent to which values were influenced by sex, age, and DXA value. Concordance of osteopenia and osteoporosis diagnoses obtained with QUS and DXA were assessed by calculation of sensitivity, specificity, and predictive values (positive predictive value: PPV, negative predictive value: NPV) [23]. Diagnoses were made using BMD T scores and the same standard thresholds (osteopenia: −2.49 ≤ BMD T score ≤ −1.01; osteoporosis: BMD T score ≤ −2.5) for both technologies. To reduce the influence of outlying measurements, quality control was applied to QUS data using threshold values previously reported with UK Biobank data ([SOS; men: ≤ 1450 and ≥ 1700 m/s, women: ≤ 1455, and ≥ 1700 m/s]; [BUA; men: ≤ 27 and ≥ 138 dB/MHz, women: ≤ 22 and ≥ 138 dB/MHz]; [BMD; men: ≤ 0.18 and ≥ 1.06 g/cm^2^, women: ≤ 0.12, and ≥ 1.025 g/cm^2^]). All analyses were conducted in the statistical environment R, with distributional regression models conducted using the GAMLSS package [24].

## Results

### Reliability

**Fig. 1:**
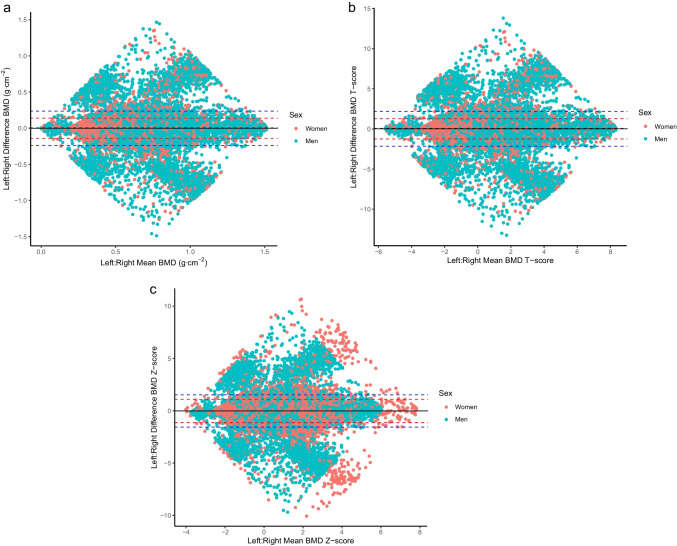
Bland-Altman plots illustrating left versus right heel quantitative ultrasound bone mineral density (BMD) values used for reliability analyses. Top left: BMD; top right: BMD T score; bottom: BMD *Z* score. Dashed black lines represent systematic bias between left and right. Dashed red lines represents typical variation for women (± 1.96*women standard deviation), dashed blue lines represents typical variation for men (± 1.96*men standard deviation)

A total set of 216,753 QUS values from the left and right heel were obtained from 194,989 participants (17,894 duplicates, 1896 triplicates 26 quadruplicates). Descriptive characteristics of the participants and summaries for the QUS BMD values are presented in Table 1. Visual representations of reliability analyses for BMD absolute scores, T scores and Z scores are presented in Bland-Altman plots in Fig. 1. The diamond shapes and corresponding points far from the central clusters illustrate large differences (e.g., errors) in left versus right values. The percentage of values beyond 1.96 standard deviations of the difference scores was, however, only 1.4% for both men and women across all three variables, demonstrating that the proportion of large differences was relatively small.

**Table 1:** Participant characteristics and summary statistics for heel quantitative ultrasound bone mineral density (BMD) measurements used for reliability analysis

Variable	Women (*n* = 116,688)	Men (*n*= 100,065)
Age (year)	57.8 (8.3)	58.7 (8.6)
Mass (kg)	71.0 (14.0)	85.4 (14.2)
Height (cm)	162.5 (6.3)	175.7 (6.8)
BMI group (%)		
Under-weight	0.9%	0.3%
Normal-weight	40.6%	26.8%
Over-weight	36.0%	49.0%
Obese	22.5%	23.9%
Heel BMD (g/cm^2^)	0.52 (0.12)	0.58 (0.14)
Heel BMD T score	−0.53 (1.08)	0.02 (1.28)

Data are provided as percentages or mean (± sd). BMD and BMD T score values are averages from left and right heel

Regression coefficients describing the typical variation in magnitude of the QUS BMD variables are presented in Table 2. The results show that sex and mean value (after controlling for sex) had similar effects when scaled, with increased variability (lower reliability) for men and higher mean values. Concordance analysis was consistent across all three QUS BMD variables showing that approximately 95% of participants were quantified in the same (~64%) or adjacent quartile (~32%), and that only ~3% were distinct by 2 quartiles and in ~1% of cases participants changed between quartiles 1 and 4 Table 3.

**Table 2:** Regression coefficients describing the effects of sex, age, and mean value on typical left versus right difference (reliability) heel quantitative ultrasound bone mineral density (BMD) values

	BMD		BMD T score		BMD Z score	
Men Sd of difference	0.12		1.10		0.78	
Women Sd of difference	0.07		0.64		0.57	
Variable	Regression coefficient (standard error)	*P* value	Regression coefficient (standard error)	*P* value	Regression coefficient (standard error)	*P* value
Intercept	−2.8 (0.002)	<0.001	−0.54 (0.002)	<0.001	−0.74 (0.002)	<0.001
Sex (men)	0.46 (0.003)	<0.001	0.50 (0.003)	<0.001	0.38 (0.003)	<0.001
Mean value	0.49 (0.003)	<0.001	0.41 (0.003)	<0.001	0.48 (0.003)	<0.001
Age	0.00 (0.003)	0.359	0.00 (0.003)	0.243	0.01 (0.003)	0.040

Regression coefficients were obtained from Gaussian distributional regression models predicting the standard deviation (sigma) using a log link function. To obtain values in the measured scale, parameters must be exponentiated. Age and mean values were centred and scaled (divided by 2*sample standard deviation) to facilitate interpretation of the intercept and compare relative importance among variables. Sex (men): represents the average difference in standard deviation between men relative to women controlling for other variables

**Table 3:** Concordance table quantifying percentage of participants categorised into quartiles based on left and right heel quantitative ultrasound bone mineral density (BMD) values

	BMD	BMD T score	BMD Z score
Concordance percentages			
Same quartile	64.3%	64.2%	64.3%
Adjacent quartile	31.7%	31.8%	31.7%
Opposite quartile (1st vs. 3rd, 2nd vs. 4th)	3.0%	3.0%	3.0%
Opposite quartile (1st vs. 4th)	1.0%	1.0%	1.0%

### Validity

A total of 5042 participants were identified, whereby at least one QUS and one DXA measurement was recorded. Data from 34 participants were subsequently removed because they were identified as outliers according to the methods described above. Descriptive characteristics of the participants and summaries for each of the variables are presented in Table 4. Consistent correlation values were obtained between QUS variables and BMD measured from DXA (Table 5). Correlations were low to modest with central estimates ranging from 0.29 to 0.44. Correlations were consistently higher for women compared with men, and for associations comprising total BMD.

**Table 4:** Participant characteristics and summary statistics for heel quantitative ultrasound (QUS) and dual-energy X-ray absorptiometry (DXA) measurements used for criterion validity analysis

Variable	Women (*n* = 2621)	Men (*n*= 2387)
Age (year)	61.2 (7.5)	62.7 (7.5)
Mass (kg)	69.6 (13.2)	84.5 (14.0)
Height (cm)	162.5 (6.3)	176.0 (6.6)
BMI group (%)		
Under-weight	1.3%	0.1%
Normal-weight	43.9%	29.3%
Over-weight	35.3%	50.5%
Obese	19.5%	20.1%
DXA total body BMD (g/cm2)	1.13 (0.13)	1.30 (0.12)
DXA Lumbar BMD (g/cm2)	1.14 (0.18)	1.26 (0.20)
DXA Femur BMD (g/cm2)	0.91 (0.14)	0.99 (0.15)
QUS BUA	74.6 (15.8)	85.7 (16.5)
QUS SOS	1550.7 (29.3)	1564.3 (31.1)
Heel BMD (g/cm2)	0.53 (0.11)	0.59 (0.12)

Data are provided as percentages or mean (± sd). *SOS* speed of sound; *BUA* broadband ultrasound attenuation. DXA Femur BMD and QUS variables represent average values from left and right side of the body

**Table 5:** Correlations between quantitative ultrasound (QUS) variables and dual-energy X-ray absorptiometry (DXA) bone mineral density (BMD) variables stratified by region and sex

	Total BMD	Lumbar BMD	Femur BMD
SOS (men)	*r* = 0.37 (0.33 to 0.42) *n* = 1250	*r* = 0.31 (0.25 to 0.35) *n* = 1249	*r* = 0.31 (0.26 to 0.36) *n* = 1261
SOS (women)	*r* = 0.44 (0.40 to 0.48) *n* = 1422	*r* = 0.38 (0.33 to 0.42) *n* = 1413	*r* = 0.37 (0.32 to 0.41) *n* = 1419
BUA (men)	*r* = 0.38 (0.33 to 0.43) *n* = 1250	*r* = 0.29 (0.24 to 0.34) *n* = 1249	*r* = 0.32 (0.27 to 0.37) *n* = 1261
BUA (women)	*r* = 0.40 (0.36 to 0.45) *n* = 1422	*r* = 0.35 (0.31 to 0.40) *n* = 1413	*r* = 0.35 (0.31 to 0.40) *n* = 1419
QUS BMD (men)	*r* = 0.39 (0.34 to 0.44) *n* = 1249	*r* = 0.31 (0.26 to 0.36) *n* = 1248	*r* = 0.33 (0.28 to 0.37) *n* = 1260
QUS BMD (women)	*r* = 0.43 (0.39 to 0.47) *n* = 1422	*r* = 0.37 (0.33 to 0.42) *n* = 1413	*r* = 0.37 (0.33 to 0.42) *n* = 1422

*SOS* Speed of sound; *BUA* broadband ultrasound attenuation

**Fig. 2:**
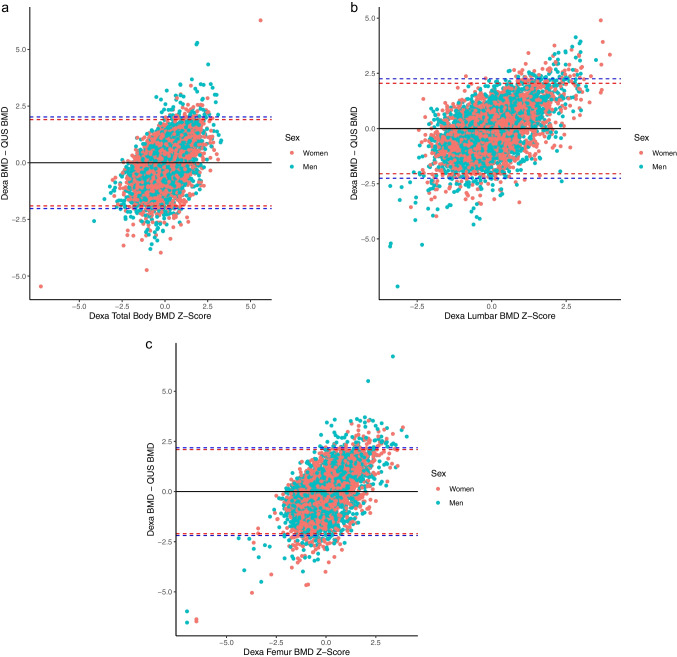
Bland-Altman plots illustrating criterion validity of standardised scores obtained with heel quantitative ultrasound (QUS) bone mineral density (BMD) and criterion dual-energy X-ray absorptiometry (DXA) BMD. Top left: DXA total body; top right: DXA lumbar; bottom: DXA femur. Dashed black lines represent systematic bias in standardised z scores between DXA and QUS. Dashed red lines represents typical variation for women (± 1.96*women standard deviation), dashed blue lines represents typical variation for men (± 1.96*men standard deviation)

A visual representation of the difference in BMD Z scores obtained with QUS and DXA are presented in Bland-Altman plots in Fig. 2. The plots revealed relatively large potential differences between measurements with consistent limits of agreement across all three DXA BMD regions. Regression coefficients describing the typical difference in BMD Z scores between the measurement tools are presented in Table 6. Analyses consistently identified greater typical differences for participants with higher DXA values. Additionally, some evidence was obtained for greater typical differences for men even after controlling for DXA values.

**Table 6:** Regression coefficients describing the effects of sex, age, and mean value on typical differences between standardised dual-energy X-ray absorptiometry (DXA) and heel quantitative ultrasound bone mineral density (BMD) values

	Total BMD vs. heel BMD Z scores		Lumbar BMD vs. heel BMD Z scores		Femur BMD vs. heel BMD Z scores	
Men Sd of difference	1.03		1.15		1.12	
Women Sd of difference	0.97		1.05		1.07	
Variable	Regression coefficient (standard error)	*P* value	Regression coefficient (standard error)	*P* value	Regression coefficient (standard error)	*P* value
Intercept	−0.17 (0.014)	<0.001	−0.13 (0.014)	<0.001	−0.11 (0.014)	<0.001
Sex (men)	0.04 (0.020)	0.076	0.06 (0.020)	0.004	0.03 (0.020)	0.134
Mean value	0.13 (0.021)	<0.001	0.14 (0.020)	<0.001	0.02 (0.021)	0.266
Age	−0.01	0.704	−0.02 (0.020	0.270	−0.01 (0.021)	0.609

Regression coefficients were obtained from Gaussian distributional regression models predicting the standard deviation (sigma) using a log link function. To obtain values in the measured scale parameters must be exponentiated. Age and mean values were centred and scaled (divided by 2*sample standard deviation) to facilitate interpretation of the intercept and compare relative importance among variables. Sex (men): represents the average difference in standard deviation between men relative to women controlling for other variables

The proportion of individuals diagnosed with osteopenia and osteoporosis, and the diagnostic comparisons between QUS and the reference DXA are presented in Table 7. Sensitivity (0.04 to 0.23) and PPV (0.13 to 0.14) were extremely low for osteoporosis diagnoses (0.34 to 4.9% of participants based on DXA values). Improved but still low performing sensitivity (0.37 to 0.62) and PPV (0.21 to 0.57) were identified for osteopenia diagnoses (7.5 to 34.1% of participants based on DXA values). In contrast, near perfect specificity (0.99) and NPV (1.0) were obtained for osteoporosis diagnoses, and high specificity (0.81 to 0.85) and PPV (0.92 to 0.98) were obtained for osteopenia diagnoses.

**Table 7:** Osteopenia and osteoporosis diagnostic comparisons between heel quantitative ultrasound and the reference dual-energy X-ray absorptiometry (DXA) bone mineral density (BMD) stratified according to region

	Diagnosis: total BMD	Diagnosis: lumbar BMD	Diagnosis: femur BMD
Osteoporosis proportion diagnosed	0.004	0.049	0.024
Osteoporosis sensitivity	0.23	0.04	0.05
Osteoporosis specificity	0.99	0.99	0.99
Osteoporosis PPV	0.14	0.26	0.13
Osteoporosis NPV	1.0	1.0	1.0
Osteopenia proportion diagnosed	0.08	0.23	0.34
Osteopenia sensitivity	0.62	0.40	0.37
Osteopenia specificity	0.81	0.83	0.85
Osteopenia PPV	0.21	0.42	0.57
Osteopenia NPV	0.98	0.94	0.92

*PPV* Positive predictive value; *NPV* negative predictive value

## Discussion

Herein, we aimed to determine whether calcaneal QUS could be used to produce reliable and informative data regarding BMD, considering the need for quicker, less expensive, less resource intensive, and less invasive methods that could be used at a population level. To achieve this, we made use of data available through the UK Biobank and first sought to determine the reliability QUS estimates of BMD using data taken from the left and right heel. Secondly, we sought to quantify agreement between BMD measurements recorded with QUS and those taken from the reference DXA. Measured in the absolute scale, QUS appeared to be reliable and most consistent for women and those with lower BMD measurements. Reliability decreased when measured in standardised scales, with large variation to be expected for BMD T scores. Similarly, when expressed in quartiles, a substantive proportion of individuals should be expected to vary between adjacent quartiles. Low to modest correlations were obtained between QUS variables and DXA BMD regardless of sex and region. These low correlations were accompanied by poor diagnostic performance, with low sensitivity and PPV for both osteopenia and osteoporosis diagnoses. Collectively, the results indicate that absolute QUS BMD data are reliable, but that these values are not likely to provide an accurate reflection of BMD of the whole body or of BMD at sites of clinical interest, such as the hip or lumbar spine. As such, using the same T score thresholds identified for DXA BMD would not seem to provide appropriate diagnostic criteria for QUS.

In this large population of middle-aged men (*n* = 100,065; aged 58.7 ± 8.6 years) and women (*n* = 116,688, aged 57.8 ± 8.3 years), assessment of reliability of QUS BMD was obtained comparing values between the left and right heel measured within the same testing session. Therefore, differences in measurements would be caused by both measurement error and true differences in BMD, which provides an upper bound determination of intra-session reliability. Given this limitation, the current study is not able to precisely quantify the actual intra-session reliability on a single heel, or indeed, the more important inter-session reliability on a single heel. From a practical perspective, however, it can be viewed that variation in QUS BMD measurements between the left and right heel should be low, such that this would indicate a stable and representative measurement of bone health. When expressed in absolute units, the standard deviation of the difference between left and right QUS BMD was equal to 0.12 g·cm^−2^ for men and 0.07 g·cm^−2^ for women indicating relatively small variation given central values of approximately 0.70 g·cm^−2^ and maximum values of approximately 1.5 g·cm^−2^. In contrast, differences between left and right heel appeared large and potentially unsuitable when expressed in standardised units. When expressed as a Z score, the standard deviation of differences was equal to 0.78 for men and 0.57 for women. From these initial values, we should expect 95% of QUS BMD Z scores obtained from the left and right heel to vary between ± 1.1 (e.g., 1.96·√2^−1^·0.78) for men and ± 0.79 (e.g., 1.96·√2^−1^·0.57) for women [25]. However, results from distributional regression analyses identified the existence of heteroscedasticity, such that variation in all QUS BMD variables between the left and right heel were influenced by both sex and average value, with greater variation for men and participants with larger BMD values. Similarly, concordance analysis casted doubt upon the reliability of QUS BMD measurements when considering participants on standardised scales. The analyses identified that a substantive proportion of individuals (~35%) should be expected to change quartile ranking based upon measurement of the left and right heel. Collectively, these results indicate that, whilst the change in absolute measurement between the left and right heel may be reasonable, BMD measurements from a relatively homogenous middle-aged population are tight enough such that variation can induce substantive differences in any ranking type of assessment.

Comparisons between QUS variables and DXA were generally consistent with those reported from several previous studies [14, 15]. Correlations obtained in the present study were slightly higher for comparisons between QUS variables and total BMD compared with comparisons that included lumbar or femur neck BMD (Table 5). Slightly higher correlations were also obtained for women compared with men. Across all analyses, however, correlations were low to modest, ranging from approximately 0.30 to 0.45. Nguyen et al. [14] reported correlations of approximately 0.35 for BUA and DXA BMD measured at the lumbar spine and femoral neck. The analyses were part of the Vietnam Osteoporosis Study comprising 1270 women and 773 men, with a mean age of approximately 45 years, but with a greater range (as low as 18-year-olds) compared to the present study. Nguyen et al. [14] proposed that the relatively low correlations were unsurprising given the fundamental differences in technologies and the differences in measurement sites, with the calcaneus comprising a lower proportion of cortical bone subjected to very different loading milieu to that of the proximal femur or lumbar spine.

In addition to investigating correlations between QUS-derived variables and DXA BMD, we also investigated differences in BMD values when the two measurement devices were placed on the same standardised scale (e.g., Z scores). Analyses identified that for both men and women, standard deviations of difference scores were approximately equal to 1.1. From these initial values we should expect that 95% of the differences in BMD Z scores between QUS and DXA would range between ± 1.5 (e.g., 1.96·√2^−1^·1.1). The upper bounds of this interval represent a large difference in the placement of a participant within a population, thus demonstrating poor criterion agreement. Additionally, analyses identified the presence of heteroscedasticity, such that those with higher DXA BMD values would experience greater variation in their QUS BMD Z scores. For example, a man with a DXA BMD Z score of 1.5 should expect standard deviation of difference scores of approximately 1.4 for total body or lumbar spine (Table 6) leading to QUS BMD Z scores expected to range between −0.4 and 3.4, further demonstrating poor agreement.

The modest correlations and large potential differences between BMD scores reported herein culminated in a poor osteopenia and osteoporosis diagnostic performance of QUS. Similar to previous studies [21], higher prevalence of osteopenia and osteoporosis were obtained using DXA BMD values from analyses at the lumbar spine and femur neck compared with the whole body. Correspondingly, higher sensitivity was obtained when using total body DXA BMD as the reference for both osteopenia (0.62) and osteoporosis (0.23) when compared with using the lumbar spine (0.40 and 0.04) or femoral neck (0.37 and 0.05). In contrast, specificity was high for both osteopenia (0.81 to 0.85) and osteoporosis (0.99). In a recent systematic review investigating QUS osteoporosis diagnostic performance in postmenopausal women, it was concluded that QUS should be considered an accurate diagnostic tool [16]. The review included 15 studies ranging from sample sizes of *n* = 43 to *n* = 1132. The mean sensitivity value was equal to 0.73 ± 0.21 and the mean specificity value equal to 0.65 ± 0.18 [16]. Most sensitivity and specificity values were, however, obtained after setting a T score threshold that optimised diagnostic performance in the reporting sample, meaning that the diagnostic performance would likely be inflated. The authors identified that diagnostic performance is likely to be influenced by the QUS device used, the prevalence of osteoporosis in the population, and that in order to achieve appropriate results, distinct T score thresholds from DXA would be required [16].

In conclusion, despite concerns that QUS and DXA measure very different qualities, QUS is routinely used and evaluated for its potential use as a diagnostic tool [6] as it represents a safer, lower cost, lower resource, and more portable alternative to DXA. The present study comprises one of the largest and most comprehensive analyses of QUS and despite the many practical advantages offered by the technology, several limitations must be acknowledged. QUS only demonstrates low to modest correlations with DXA BMD values; however, researchers have identified that correlations may be influenced by the specific QUS and DXA scanner comparison as there are no studies that provide standardized equations such as those that exist between major DXA manufacturers [26, 27]. In addition, reliability of QUS BMD measurements may be limited, especially for men exhibiting larger values, or when results are expressed in standardised scales such as Z scores, T scores, or quartiles. In addition, osteopenia and osteoporosis diagnostic performance of QUS may be limited, depending upon a range of factors including prevalence in the population. In order to achieve appropriate diagnostic performance, research suggests that development of specific threshold values is required.
